# Bloody noise: The impact of blood‐flow artifacts on registration

**DOI:** 10.1002/hbm.26426

**Published:** 2023-07-29

**Authors:** John D. Lewis, Vladimir S. Fonov, D. Louis Collins

**Affiliations:** ^1^ The Hospital for Sick Children University of Toronto Toronto, ON Canada; ^2^ Montreal Neurological Institute McGill University Montreal Canada

**Keywords:** ABIDE, blood suppression, blood‐flow artifacts, registration

## Abstract

Blood‐flow artifacts present a serious challenge for most, if not all, volumetric analytical approaches. We utilize T1‐weighted data with prominent blood‐flow artifacts from the Autism Brain Imaging Data Exchange (ABIDE) multisite agglomerative dataset to assess the impact that such blood‐flow artifacts have on registration of T1‐weighted data to a template. We use a heuristic approach to identify the blood‐flow artifacts in these data; we use the resulting blood masks to turn the underlying voxels to the intensity of the cerebro‐spinal fluid, thus mimicking the effect of blood suppression. We then register both the original data and the deblooded data to a common T1‐weighted template, and compare the quality of those registrations to the template in terms of similarity to the template. The registrations to the template based on the deblooded data yield significantly higher similarity values compared with those based on the original data. Additionally, we measure the nonlinear deformations needed to transform the data from the position achieved by registering the original data to the template to the position achieved by registering the deblooded data to the template. The results indicate that blood‐flow artifacts may seriously impact data processing that depends on registration to a template, that is, most all data processing.

## Graphical abstract

Blood‐flow artifacts present a serious challenge for most, if not all, volumetric analytical approaches, and even surface‐based approaches. We utilize T1‐weighted data with prominent blood‐flow artifacts from the Autism Brain Imaging Data Exchange (ABIDE) multisite agglomerative dataset to assess the impact that such blood‐flow artifacts have on registration of T1‐weighted data to a template. We use a heuristic approach to identify the blood‐flow artifacts in these data; we then use the resulting blood masks to mimick the effect of blood suppression. The registrations to the template based on the deblooded data yield significantly higher similarity values compared with those based on the original data. The results indicate that blood‐flow artifacts may seriously impact data processing that depends on registration to a template, that is, most all data processing.

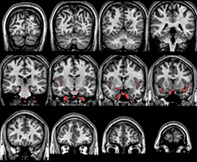

## 1 INTRODUCTION

Magnetic resonance imaging (MRI) scans can contain many different types of artifacts, such as motion artifacts, inhomogeneities, magnetic susceptibility artifacts, and artifacts due to blood flow. All of these can interfere with processing the data, and so with the veracity of the results. Some of these artifact types have no realistic solution, for example, magnetic susceptibility artifacts due to metallic dental implants; some have partial solutions in the form of special sequences, for example, motion correction (Godenschweger et al., 2016); and some can be dealt with in postprocessing, for example, inhomogeneity artifacts (Vovk et al., 2007). Artifacts due to blood flow can now largely be eliminated via the use of sequences that are sensitive to local motion. But blood suppression when using 3D volumetric imaging techniques has proven difficult (Crowe et al., 2006) and though much improved in recent years, is still an active area of research, and such data may still show artifacts of incomplete suppression; and legacy datasets may show little or no blood suppression.

Here, we investigate the impact of the presence of such blood‐flow artifacts on one of the core steps in processing data for most, if not all, types of analyses: registration of the T1‐weighted scan to a common template. Blood‐flow artifacts can interfere with the accurate identification of a brain mask, which is crucial for registration. The conflict between bright blood voxels in the subject and dark blood voxels in a template based on data with proper blood suppression can interfere with registration algorithms based on optimization of image similarity. For linear registration, this can result in registrations that are rotated, translated, or scaled compared with what they would be without the blood‐flow artifacts. For nonlinear registrations, blood‐flow artifacts can result in distortions that align a blood vessel in the subject with a gyrus in the template, causing local displacements of several millimeters, adding to the misalignment problems caused by the linear registration. Registration is central to voxel‐based morphometry (VBM) analyses, (volumetric) functional MRI (fMRI) analyses, and any other approaches that compare values across voxels across subjects; it is also important for tools that utilize information stored in a template to, for example, guide tissue classification, identify brain anatomy, guide surface construction, and so forth. Thus, understanding the role of blood‐flow artifacts in interfering with accurate registration is crucial.

We use the T1‐weighted data with blood‐flow artifacts from the Autism Brain Imaging Data Exchange (ABIDE) multisite agglomerative dataset^1^ to study this issue. The ABIDE data were collected independently at more than 24 international brain imaging laboratories, using scanners from various manufacturers, and protocols that were developed without coordination across sites. The scans from several of these sites show prominent blood‐flow artifacts, perhaps due to a failure to take full advantage of the scanner options available to suppress blood‐flow artifacts, or because such options were inadequate to do so. We describe a heuristic approach to identify the blood‐flow artifacts in these scans, using a blood probability map constructed from magnetic resonance angiography (MRA) scans from the BrainWeb dataset.^2^ Our approach uses tissue classification, gradient information, and the blood probability map to construct a blood mask; we then replace the values underlying that blood mask with the mean value of cerebrospinal fluid (CSF), mimicking the result that would be achieved with well‐functioning blood suppression; we refer to this as the *deblooded* version of the scan.

To test the impact of the presence of the blood‐flow artifacts on registration of the T1‐weighted scans to a common template, we registered both the original scans and the deblooded scans to the common template, and for each pair, measured the similarity of the two transformed scans to the template. We also measured the deformations required to nonlinearly transform the original scan overlayed on the template to the deblooded scan overlayed on the template in order to show the differences in the nonlinear deformations caused by the blood‐flow artifacts.

Beyond showing the impact of blood‐flow artifacts on registration, we also provide our blood masks and deblooded data^3^ for the use of the neuroimaging community in the hope that this will help researchers produce more accurate results on the ABIDE data, and that it might serve as training data for attempts to train a neural network to identify blood‐flow artifacts, for use with other datasets.

## 2 MATERIALS AND METHODS

### 2.1 Participants

**FIGURE 1:**
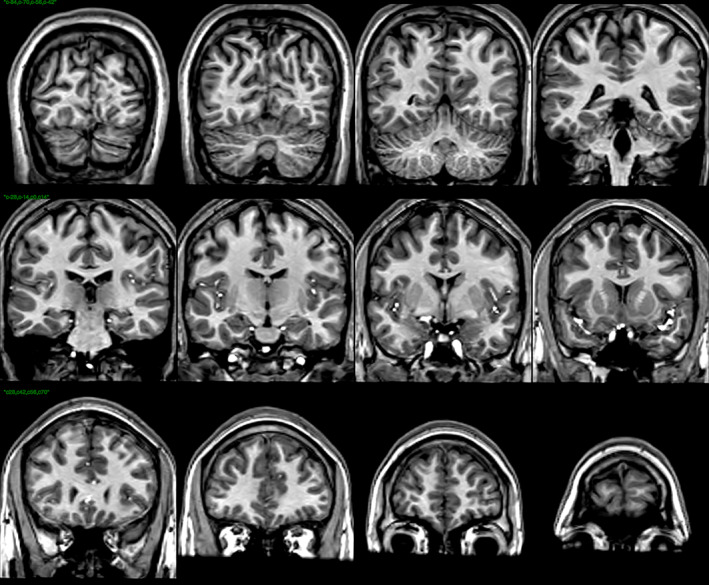
Coronal views of the T1‐weighted scan for one subject, as an example. Notice the bright blood‐flow artifacts in the Sylvian and Longitudinal Fissures and in inferior regions. Such blood‐flow artifacts are problematic for registration.

This study uses all of the ABIDE data for which the T1‐weighted data were collected without successful blood suppression. There were 1064 such subjects. An example scan from one such site is shown in Figure 1. The demographic details for those sites are shown in Table 1. The scanner manufacturers and models for each site, and the T1‐weighted scan resolutions are listed in Table 2.

**TABLE 1:** Demographics.

Site	No. of subjects	Age	Male/female	ASD/CTRL
KKI	266	Range: [8–13]	182/84	188/78
NYU	289	Range: [6.5–39.1]	242/47	154/135
OHSU	121	Range: [7–15.2]	85/36	71/50
PITT	57	Range: [9.3–35.2]	49/8	30/27
UCLA	141	Range: [8–17.9]	122/19	78/63
USM	134	Range: [8.8–50.2]	129/5	75/59
YALE	56	Range: [7–17.8]	40/16	28/28

Abbreviations: KKI, Kennedy Krieger Institute; NYU, New York University; OHSU, Oregon Health and Science University; PITT, University of Pittsburgh School of Medicine; UCLA, University of California Los Angeles; USM, Utah School of Medicine; Yale School of Medicine.

**TABLE 2:** Multisite scanner and scan resolution info.

Site	Scanner	TE	TI	TR	FA	Resolution
KKI	Philips 3 T Achieva	3.7	1000	3000	8	1 mm × 1 mm × 1 mm
NYU	Siemens 3 T Allegra	3.25	1100	2530	7	1.3 mm × 1.0 mm × 1.3 mm
OHSU	Siemens 3 T Tim Trio	3.58	900	2300	10	1 mm × 1 mm × 1.1 mm
PITT	Siemens 3 T Allegra	3.93	1000	2100	7	1.1 mm × 1.1 mm × 1.1 mm
UCLA	Siemens 3 T Tim Trio	2.86	853	2300	9	1 mm × 1 mm × 1.2 mm
USM	Siemens 3 T Tim Trio	2.91	900	2300	9	1 mm × 1 mm × 1.2 mm
YALE	Siemens 3 T Magnetom	1.73	624	1230	9	1 mm × 1 mm × 1 mm

Abbreviations: 3 T, 3‐Tesla; FA, Fractional Anisotropy; KKI, Kennedy Krieger Institute; NYU, New York University; OHSU, Oregon Health and Science University; PITT, University of Pittsburgh School of Medicine; TE, Echo Time; TI, Inversion Time; TR, Repetition Time; UCLA, University of California Los Angeles; USM, Utah School of Medicine; Yale School of Medicine.

### 2.2 Blood‐flow artifact removal

**FIGURE 2:**
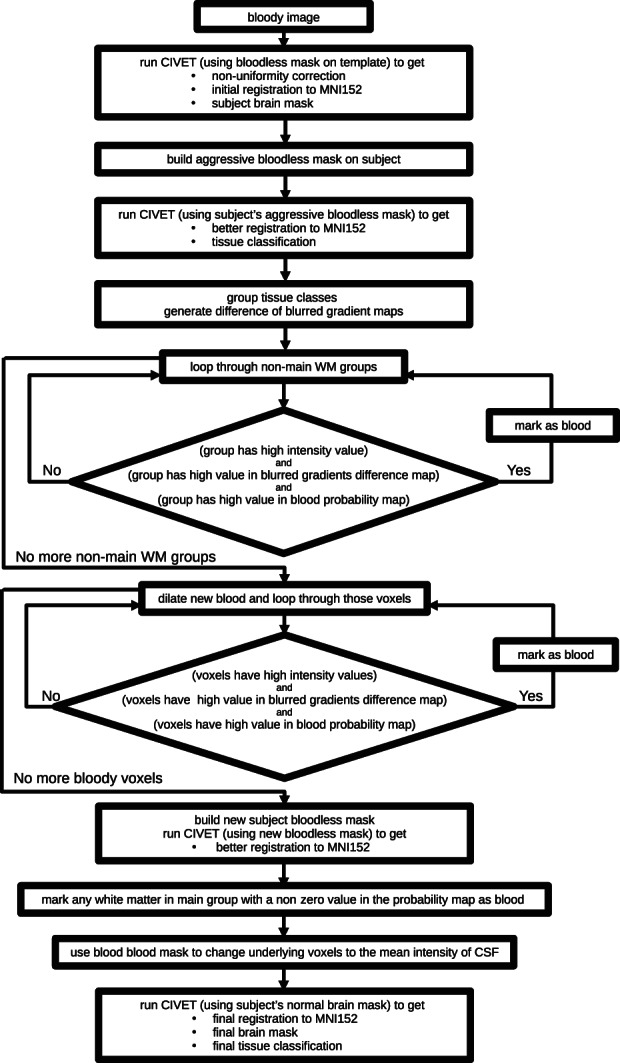
A flow chart of the blood‐removal process.

**FIGURE 3:**
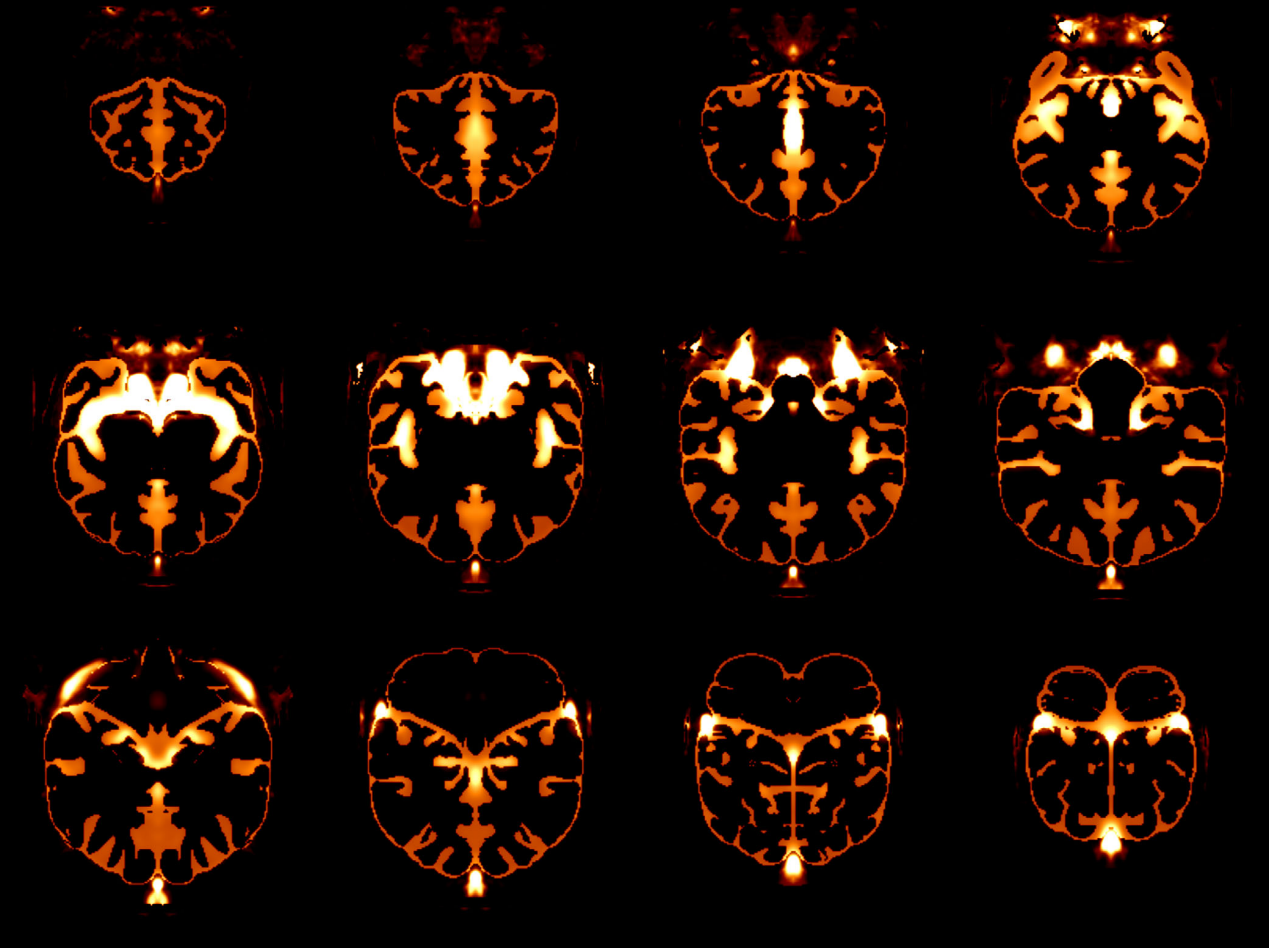
Coronal views of the blood probability map on the template. The map is shown on the hotmetal scale, that is, the highest probabilities are white.

**FIGURE 4:**
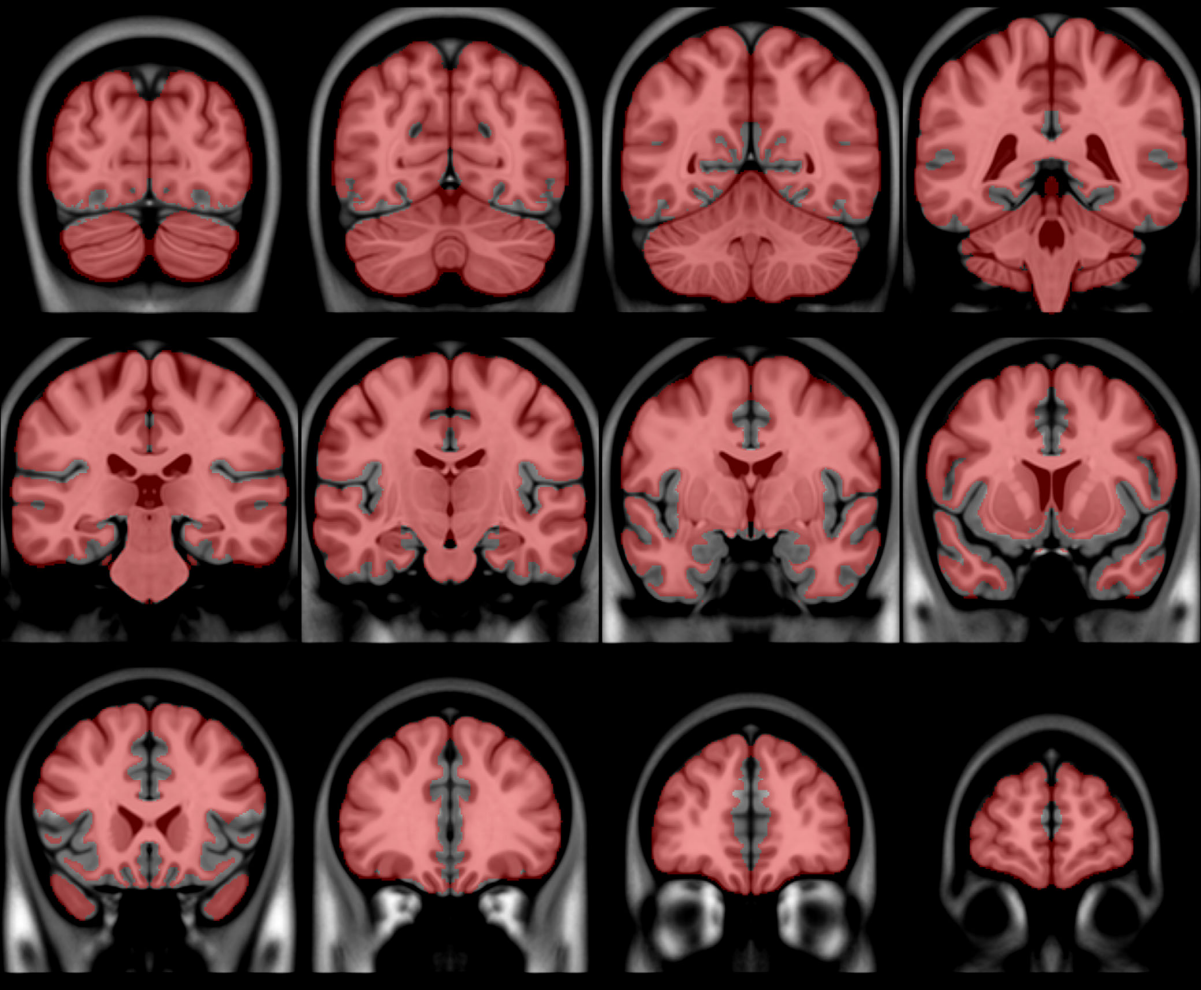
Coronal views of the bloodless mask on the template. The mask is shown in red, with enough transparency to show the underlying structure. Note that it does not include the regions with a high probability of having blood vessels.

The blood‐flow artifacts in these scans cause a variety of problems for processing. In order to eliminate these problems, to the extent possible, we have developed a method to remove these blood‐flow artifacts from the data. A flowchart of our method is shown in Figure 2. Our method relies on registration; but, as noted above, registration is impacted by blood‐flow artifacts. To lessen the impact that the blood‐flow artifacts have on registration, the initial registrations were done using masks meant to ignore the blood‐flow artifacts. The first such mask was for the template, that is, the fixed volume. This mask was based on a blood probability map (constructed from MRA data). The MRA data were from the BrainWeb dataset^4^ (Aubert‐Broche et al., 2006). This dataset consists of 20 brain scans acquired on a 1.5 T Siemens Sonata Vision clinical scanner (Siemens Medical Systems, Erlangen, Germany). The protocol comprises conventional whole‐head high‐resolution T1‐, T2‐, and proton density‐weighted scans, and an MRA scan. Each subject's MRA scan was linearly registered to their T2‐weighted scan, and each T2‐weighted scan was linearly and then nonlinearly registered to the MNI152 T2‐weighted template (Fonov et al., 2009). Linear registration was done using *bestlinreg.pl* (Dadar et al., 2018). Nonlinear registration was done using *antsRegistration* (Avants et al., 2011). The transform determined to register the subject's T2‐weighted scan to the MNI152 T2‐weighted template was concatenated to the transform to register the subject's MRA scan to their T2‐weighted scan, and that transform was used to transform the MRA data to overlay the MNI152 T2‐weighted template; the transformed MRA scans were then averaged and the result normalized to form the blood probability map. This blood probability map is shown in Figure 3. This blood probability map was thresholded (at 0.1) and combined with the brain mask of the MNI152 template (Fonov et al., 2009) to form an initial roughly bloodless brain mask that excluded the areas that were most likely to contain blood vessels. The threshold of 0.1 was chosen by exploring various values, and choosing one that would best eliminate most of the blood vessels, but leave the most nonblood brain tissue. This initial bloodless mask is shown in Figure 4.

This bloodless mask was used in an initial attempt to register an individual's T1‐weighted data to the MNI152 T1‐weighted template (Fonov et al., 2009). Linear registration was done using *bestlinreg.pl* (Dadar et al., 2018). Nonlinear registrations were done using *antsRegistration* (Avants et al., 2011) with the mutual information metric. This initial registration was imperfect, but sufficient to provide a reasonable alignment between the subject's T1‐weighted scan and the blood probability map, that is, to approximately register the subject's T1‐weighted scan to the MNI152 T1‐weighted template.

A brain mask for the subject was then extracted using *mincbet* and intersected with a binarized copy of the blood probability map thresholded at 0.2 to exclude the major blood‐flow artifacts from the mask. The threshold of 0.2 was again chosen by exploring various values, and choosing one that would best eliminate the major blood vessels, but only those vessels. With this mask the subject's T1‐weighted volume was subjected to nonuniformity correction (Sled et al., 1998) and intensity normalization, and once again registered to the MNI152 T1‐weighted template to achieve a better registration. Finally, the blood‐flow artifacts were identified by a heuristic process, to produce blood masks on the subject, after which the voxels underlying the blood masks were set to the mean intensity of the CSF. The heuristics are:

1. voxels with a high value in the blood probability map are more likely to be blood‐flow artifacts;
2. bright voxels that are outside of the main body of white matter are potentially blood‐flow artifacts;
3. voxels with strong gradients in nearly‐adjacent voxels are potentially blood‐flow artifacts; and
4. voxels that are adjacent to blood‐flow artifacts are potentially also blood‐flow artifacts, that is, blood is not seen in isolated voxels, rather it is seen in a vascular structure.

Tissue classification was performed on the subject's nonuniformity corrected T1‐weighted data (Tohka et al., 2004). White matter voxels were then grouped with *mincmorph* based on connectivity, with the main group being the largest. Note that true white matter should all form a single connected group. But, in fact, thin strands of white matter, for example, in narrow gyri, might fluctuate in intensity sufficiently to form separate groups of white matter in the tissue classification. Also blood‐flow artifacts might be included as part of the main white‐matter group (e.g., where blood vessels wrap the anterior of the corpus callosum), or might constitute one of the other white‐matter groups. And a white‐matter group that is not part of the main white‐matter group is quite possibly a blood‐flow artifact.

**FIGURE 5:**
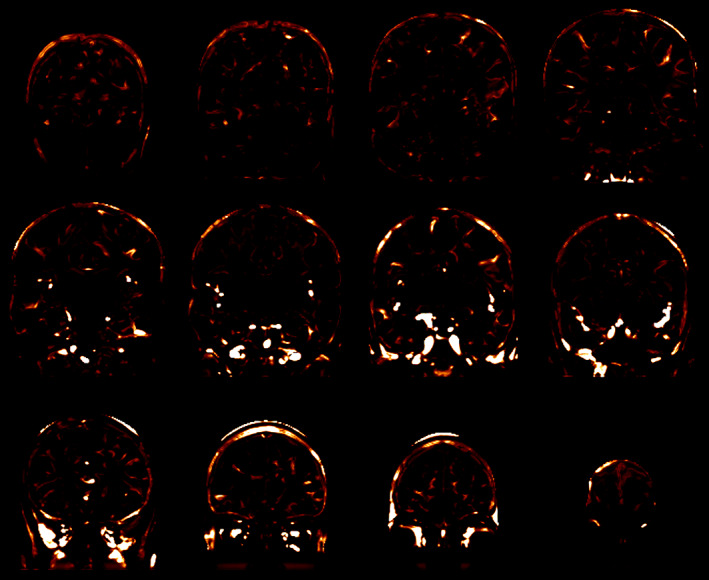
Coronal views of the blurred gradient difference map corresponding to the T1‐weighted scan shown in Figure 1. The map is shown on the hotmetal scale. Note that it highlights the blood‐flow artifacts quite well. Nonblood voxels with high values in this blurred gradient difference map are not classified as blood‐flow artifacts if they do not satisfy the other heuristics.

A gradient map was created, using *mincblur ‐gradient ‐fwhm 1*, from the subject's nonuniformity corrected T1‐weighted scan and another from the MNI152 T1‐weighted template. The gradient map for the MNI152 T1‐weighted template was subtracted from the gradient map for the subject's nonuniformity corrected T1‐weighted scan, to eliminate, to the extent possible, those gradients at the edge of white matter. The gradient difference map was then blurred with *mincblur ‐fwhm 2* to strengthen those areas where the gradients were due to blood‐flow in vessels, and weaken those gradients that were not. Thus, voxels with strong gradients in this blurred gradient difference map were quite possibly due to blood‐flow artifacts. An example of such a blurred gradient difference map is depicted in Figure 5.

For the voxels classified as white‐matter tissue that are outside of the main body of the white matter, the blurred gradient difference map together with the blood probability map were used to determine which voxels should be classified as blood‐flow artifacts. The voxels that were classified as blood‐flow artifacts were then used as a starting point to expand blood‐flow artifacts outward in the vascular system, in order to more completely identify blood vessels. This was done simply by iteratively dilating the set of voxels classified as blood‐flow artifacts, and then evaluating whether the voxels newly classified as potential blood‐flow artifacts met the criteria for this classification, that is, they were classified as white‐matter tissue, but are not grouped together with the main group of white‐matter voxels, and they have relatively high values in both the blurred gradient difference map and in the blood probability map.

The initial brain mask might contain some extra voxels on its periphery, due to blood‐flow artifacts. These extra voxels are removed once the blood‐flow artifacts have been identified. At intermediate stages, the subject's brain mask minus the set of voxels that have thus far been classified as blood‐flow artifacts constitute a mask that more accurately excludes most blood‐flow artifacts. This new mask is used to refine the original registration to the template, using it rather than the original so‐called bloodless mask based on the highest blood probabilities in the blood probability map. The result now allows the blood‐flow artifacts that were grouped together with the main set of white‐matter voxels to be identified as blood‐flow artifacts, due to those voxels falling in places with high values in the blood probability map.

**FIGURE 6:**
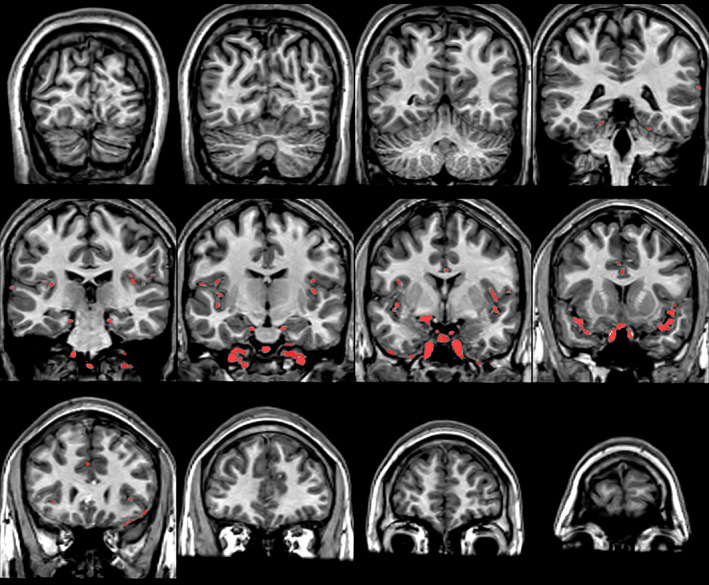
Coronal views of the blood mask for that subject. Notice that the bright blood‐flow artifacts in the Sylvian and Longitudinal Fissures and in inferior regions, for the most part, have been identified.

The resulting set of voxels that have been classified as blood‐flow artifacts constitute a blood mask. Figure 6 shows the blood mask for the T1‐weighted scan shown in Figure 1 overlaid on the original T1‐weighted scan. Notice that the blood mask identifies most of the blood‐flow artifacts in the Sylvian and Longitudinal Fissures and in inferior regions, but it is not perfect. The method is conservative in that it does not identify as blood‐flow artifacts those voxels that do not have sufficient intensity, or lack the surrounding gradients, or are in a position that has a low probability of being blood. Thus, there may be voxels that can be identified by the eye as blood, but are not identified as blood by the method. But, recall that the goal is not perfect identification of blood‐flow artifacts; rather it is to solve the problem that blood‐flow artifacts cause. Note that the blood mask does not need to be perfect to improve the registration. Minor residual blood‐flow artifacts can be tolerated. But, turning true white matter to the intensity of CSF can be catastrophic. The method is designed to ensure that it never identifies as blood‐flow artifact any voxels that are not. This was verified via manual inspection of all 1064 scans; every place that was identified as blood‐flow artifact was inspected to ensure that the corresponding voxels in the T1‐weighted volume were indeed blood‐flow artifacts.

**FIGURE 7:**
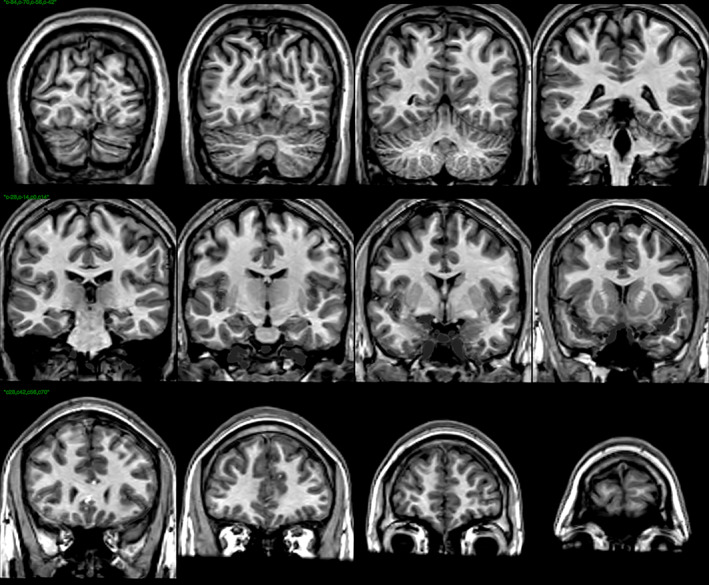
Coronal views of the deblooded t1‐weighted data for that subject. Notice that the bright blood‐flow artifacts in the Sylvian and Longitudinal Fissures and in inferior regions, for the most part, are no longer visible.

This blood mask is then used to set the underlying voxels to the mean value of the intensity of the voxels classified as CSF. This is done by dilating the blood mask, and then blurring it, and then setting the underlying voxels to their original value multiplied by one minus the value of the blurred dilated blood mask plus the mean value of CSF multiplied by the value of the blurred dilated blood mask, that is, by smoothy inserting the mean value of CSF in place of the values of the voxels identified as blood‐flow artifacts. The result is what we refer to as the deblooded scan. Figure 7 shows the deblooded T1‐weighted data corresponding to the T1‐weighted scan shown in Figure 1.

### 2.3 Analysis

Each subject's original (bloody) scan, after nonuniformity correction, was registered to the MNI152 T1‐weighted template, first linearly, using *bestlinreg* (Dadar et al., 2018) and then nonlinearly using *antsRegistration* (Avants et al., 2011) with the mutual information metric. Let us refer to the resulting linear transform as $T_{\mathrm{lin}}^{\text{bloody}}$. Let us refer to the resulting concatenated linear + nonlinear transform as $T_{\text{linnl}}^{\text{bloody}}$. The same was done for the deblooded version of each scan. Let us refer to the resulting linear transform as $T_{\mathrm{lin}}^{\text{deblooded}}$, and the resulting concatenated linear + nonlinear transform as $T_{\text{linnl}}^{\text{deblooded}}$. The transforms resulting from those registrations were applied to their respective scans, and their associated masks. We then assessed how well the versions based on registrations using the original (bloody) data aligned to the template and how well the versions based on registrations using the deblooded data aligned to the template. Let us refer to the transformed version of the brain mask for the original scan using $T_{\mathrm{lin}}^{\text{bloody}}$ as $T_{\mathrm{lin}}^{\text{bloody}}\left(\text{bloody}\_\text{mask}\right)$ and the transformed version of the deblooded scan using $T_{\mathrm{lin}}^{\text{bloody}}$ as $T_{\mathrm{lin}}^{\text{bloody}}\left(\text{deblooded}\right)$; and the transformed version of the brain mask for the deblooded version of the scan using $T_{\mathrm{lin}}^{\text{deblooded}}$ as $T_{\mathrm{lin}}^{\text{deblooded}}\left(\text{deblooded}\_\text{mask}\right)$ and the transformed version of the deblooded scan using $T_{\mathrm{lin}}^{\text{deblooded}}$ as $T_{\mathrm{lin}}^{\text{deblooded}}\left(\text{deblooded}\right)$. And let us refer to the transformed versions based on $T_{\text{linnl}}^{\text{bloody}}$ as $T_{\text{linnl}}^{\text{bloody}}\left(\text{bloody}\_\text{mask}\right)$ and $T_{\text{linnl}}^{\text{bloody}}\left(\text{deblooded}\right)$; and the transformed versions based on $T_{\text{linnl}}^{\text{deblooded}}$ as $T_{\text{linnl}}^{\text{deblooded}}\left(\text{deblooded}\_\text{mask}\right)$ and $T_{\text{linnl}}^{\text{deblooded}}\left(\text{deblooded}\right)$. We assessed the alignment to the template in two ways: using the Dice Kappa Overlap ratio metric to measure how well the subject's brain mask aligns with the template mask; and the Mutual Information similarity metric to measure the similarity of the transformed volumes to the template. That is, we assessed the Dice Kappa Overlap ratio of $T_{\mathrm{lin}}^{\text{bloody}}\left(\text{bloody}\_\text{mask}\right)$ to the MNI152 template mask; and the Dice Kappa Overlap ratio of $T_{\mathrm{lin}}^{\text{deblooded}}\left(\text{deblooded}\_\text{mask}\right)$ to the MNI152 template mask; and the similarity of $T_{\mathrm{lin}}^{\text{bloody}}\left(\text{deblooded}\right)$ to the MNI152 template; and the similarity of $T_{\mathrm{lin}}^{\text{deblooded}}\left(\text{deblooded}\right)$ to the MNI152 template. We assessed the concatenated linear + nonlinear transforms in the same way. Note that for the similarity measures, the volumes being compared are always transformed versions of the same MRI, that is, the deblooded version of the original MRI. Thus, it is really the impact of the blood‐flow artifacts on the transforms that is being measured. We then collected all of the results, and assessed the differences between the similarity results for the registrations based on the original (bloody) data and those based on the deblooded data; in all four cases, this was done with a paired *t*‐test.

We then asked what these differences in similarity meant in term of the distortions the presence of blood‐flow artifacts in the scans imposed on the transformed data. We did this by nonlinearly registering $T_{\text{linnl}}^{\text{bloody}}\left(\text{deblooded}\right)$ to $T_{\text{linnl}}^{\text{deblooded}}\left(\text{deblooded}\right)$ and then assessing the warps in the resulting nonlinear transform. We suggest that all such warping will be due to the presence of blood‐flow artifacts, either because of their effects on brain extraction, on nonuniformity correction, or on registration. We assessed these nonlinear transforms in two ways. To assess the local compression or expansion of voxels, we took the absolute value of the determinant of the transform. We then took the maximum value in each voxel across subjects to create a map of where compression or expansion occurred. But, the nonlinear transforms may also involve local translations, in which voxels shift with minimal expansion or compression. In order to assess that part of the nonlinear transforms, we measured the magnitude of the displacements. We took the maximum value across subjects for each voxel to create a map of where such displacements occurred.

## 3 RESULTS

Table 3 presents:

1. The means and variances for the Dice Kappa Overlap ratio metrics for $T_{\mathrm{lin}}^{\text{bloody}}\left(\text{bloodymask}\right)$ and for $T_{\mathrm{lin}}^{\text{deblooded}}\left(\text{debloodedmask}\right)$, and the significance of the difference between the two;
2. The means and variances for the similarity metrics for $T_{\mathrm{lin}}^{\text{bloody}}\left(\text{deblooded}\right)$ and for $T_{\mathrm{lin}}^{\text{deblooded}}\left(\text{deblooded}\right)$, and the significance of the difference between the two;
3. The means and variances for the Dice Kappa Overlap ratio metrics for $T_{\text{linnl}}^{\text{bloody}}\left(\text{bloodymask}\right)$ compared with the MNI152 mask, and for the MNI152 mask compared with $T_{\text{linnl}}^{\text{deblooded}}\left(\text{debloodedmask}\right)$, and the significance of the difference between the two;
4. The means and variances for the similarity metrics for $T_{\mathrm{lin}}^{\text{bloody}}\left(\text{deblooded}\right)$ and for $T_{\mathrm{lin}}^{\text{deblooded}}\left(\text{deblooded}\right)$, and the significance of the difference between the two.

**TABLE 3:** Comparison of $T_{\mathrm{lin}}^{\text{bloody}}$ and $T_{\mathrm{lin}}^{\text{deblooded}}$; and of $T_{\text{linnl}}^{\text{bloody}}$ and $T_{\text{linnl}}^{\text{deblooded}}$.

	Original (bloody)	Deblooded	Significance
	Mean ± variance	Mean ± variance	
Linear mask dice	0.939±9.86e−04	0.953±2.82e−04	1.37e−51
Linear similarity (MI)	0.014±6.11e−04	0.019±7.25e−05	2.35e−12
Nonlinear mask dice	0.965±9.06e−04	0.979±1.94e−04	5.19e−50
Nonlinear similarity (MI)	0.054±5.74e−02	0.070±3.14e−04	3.09e−02

*Note*: The mask comparisons use the Dice Kappa Overlap ratio metric; the volume comparisons use voxel‐wise similarity with mutual information. Note that the transforms based on the deblooded versions of the scans yield higher Dice Kappa Overlap measures and similarity scores for both the linear and nonlinear transforms.

In all cases, the significance is from a two‐tailed paired *t*‐test. Notice that the similarity metrics are always comparing the deblooded scan to the MNI152 template, so it is really the transforms that are being compared. Notice that the mean similarity for the deblooded data is greater than that of the original (bloody) data, and the difference is highly significant.

**FIGURE 8:**
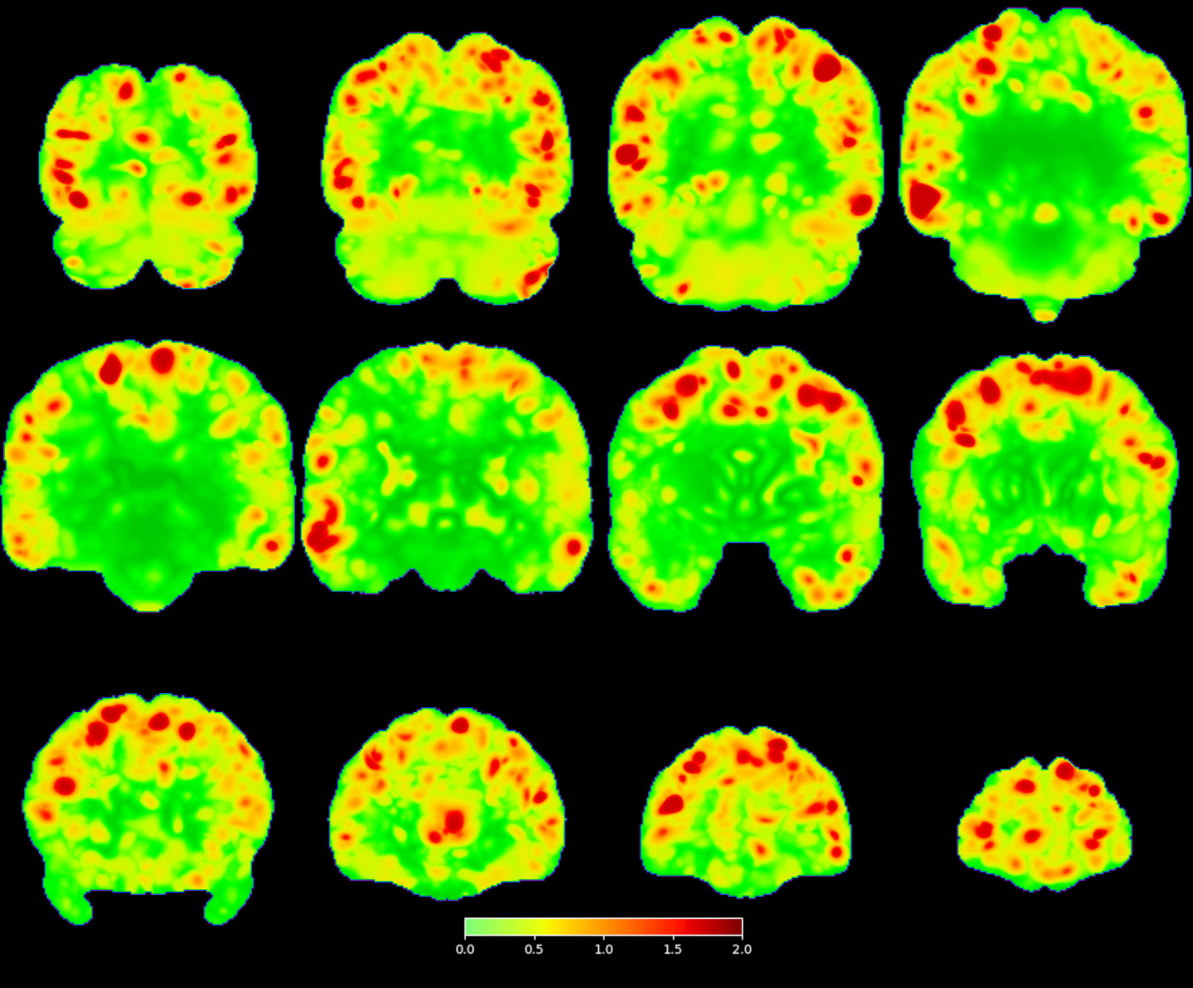
Coronal views of the maximum absolute Jacobian determinant, across subjects, of the transformations found by registering the scans overlayed on the template with the bloody transformations on their counterparts overlayed on the template with the deblooded transformations.

**FIGURE 9:**
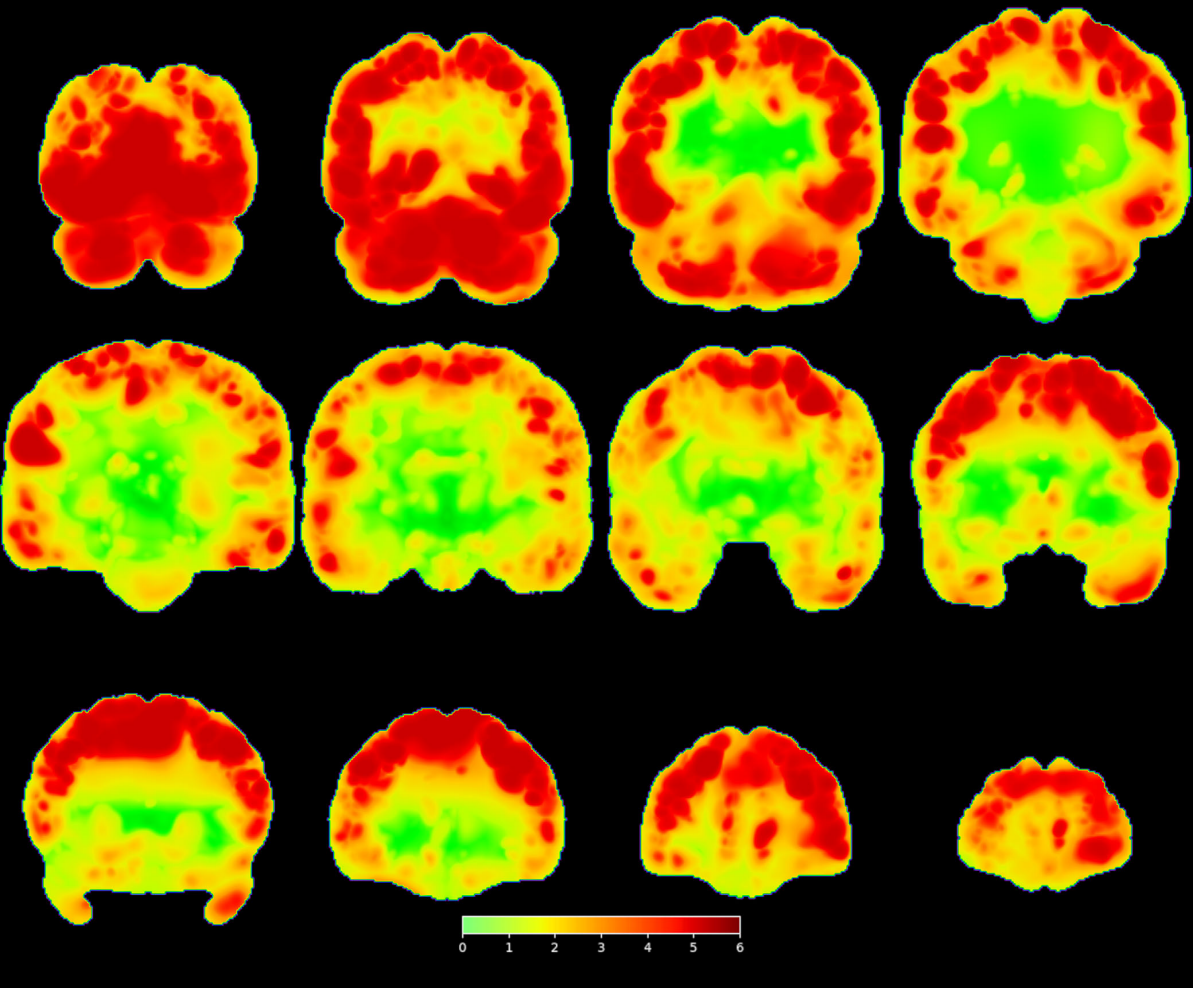
Coronal views of the maximum displacement of each voxel, across subjects, calculated from the transformations found by registering the scans overlayed on the template with the bloody transformations on their counterparts overlayed on the template with the deblooded transformations across subjects.

The warpings required to transform the scans registered to the template based on the original (bloody) data to the scans registered to the template based on the deblooded scans are shown in Figures 8 and 9. Figure 8 shows the maximum of the absolute value of the determinant across all 1064 subjects at each voxel. The voxels shown in red have values >2, and so represent substantial local expansion or compression in the scans. Notice that such voxels are seen over much of the cortex outside of the central white matter, that is, amongst the sulci and gyri.

Figure 9 shows the maximum of the magnitude of the displacement of each voxel across all 1064 subjects. The voxels shown in red have values >5, and so represent substantial local spatial displacement in the scans. Notice that again such displacements are seen over much of the brain outside of the central white matter, that is, amongst the sulci and gyri.

## 4 DISCUSSION

In this article, we have investigated the impact of blood‐flow artifacts on one of the core steps in processing scans for volumetric fMRI analyses, VBM, or most any other type of analysis, including surface‐based analyses. We used T1‐weighted data from the frequently analyzed ABIDE multisite agglomerative dataset, several sites of which show this artifact. We described a heuristic approach to identify the blood‐flow artifacts in these scans, using a blood probability map constructed from MRA scans from the BrainWeb dataset. Our approach used tissue classification, gradient information, and the blood probability map to construct a blood mask; and then replaced the values underlying that blood mask with the mean value of CSF, mimicking the result that would be achieved with well‐functioning blood suppression. We then registered both the original scan and the deblooded scan to the MNI152 T1‐weighted template, and applied both the resulting transforms to the deblooded scan. We then measured the similarity of either to the MNI152 T1‐weighted template, thus measuring the impact of the blood‐flow artifacts on the registration. We stress that we compared the similarity of the MNI152 T1‐weighted template to the deblooded scan overlaid on it using either the transform arrived at by registering the original bloody scan to it, or the transform arrived at by registering the deblooded scan to it; thus the comparison is only of the transforms, since in both cases, the deblooded scan is what is transformed. So, this provides a quantitative assessment of the quality of the registration. The results showed that the registrations performed with the deblooded version of the scans yielded significantly greater similarity than the registrations performed with the original version of the scans. We then measured the deformations required to bring the volume registered to the template with the original scan to overlay the volume registered to the template with the deblooded version. The results showed that those distortions occurred in regions of sulci and gyri, and that they were widely distributed, occurring in both hemispheres, and in all lobes.

These results indicate that such blood‐flow artifacts may seriously impact data processing that depends on registration to a template, that is, most all data processing. These results also indicate that previous studies using these data without dealing with the blood‐flow artifacts may need to be reconsidered.

It should be noted that the results reported here are based on our standard approach to image registration, with our standard settings. It may be that different approaches to registration are more robust against these sorts of blood‐flow artifacts. These results are also based only on the blood‐flow artifacts seen in the ABIDE data. It may be that different results might be found in data with blood‐flow artifacts produced by other parameter settings or acquired on different scanners.

In the hope that this work will inspire others to take the presence of blood‐flow artifacts in the ABIDE data into account, we provide all of our blood masks for the use of the neuroscience community. The masks can be found on G‐Node at https://gin.g-node.org/johndlewis/BloodyNoise/ABIDE/. We also provide our scripts for identifying these blood artifacts, so that others can use them on other datasets that show such artifacts. The scripts can be found at https://gin.g-node.org/johndlewis/BloodyNoise/code/. Unfortunately, one of the limitations of this work is that our heuristic approach is extremely computationally expensive. Future research should strive to solve this problem, so that if datasets other than these show such artifacts, deblooded versions of the scans can be efficiently produced. Our hope is that the blood masks provided will be useful for such work, possibly providing the training data for a deep‐learning approach.

## CONFLICT OF INTEREST STATEMENT

The authors declare no conflicts of interest.

## Data Availability

The ABIDE data are available at https://fcon_1000.projects.nitrc.org/indi/abide/. The data, with the blood‐flow artifacts removed, as well as the blood masks, will be available at https://gin.g-node.org/johndlewis/BloodyNoise/ABIDE/. The scripts for identifying and eliminating these blood‐flow artifacts will be available at https://gin.g-node.org/johndlewis/BloodyNoise/code/. The MRA data are available at https://brainweb.bic.mni.mcgill.ca/brainweb/.

## Footnotes

^1^

https://fcon_1000.projects.nitrc.org/indi/abide/.

^2^

https://brainweb.bic.mni.mcgill.ca/brainweb/.

^3^

https://gin.g-node.org/johndlewis/BloodyNoise/ABIDE.

^4^

https://brainweb.bic.mni.mcgill.ca/brainweb/.
